# An account of the Ebola virus disease outbreak in Nigeria: implications and lessons learnt

**DOI:** 10.1186/s12889-017-4535-x

**Published:** 2017-07-10

**Authors:** Akaninyene Otu, Soter Ameh, Egbe Osifo-Dawodu, Enoma Alade, Susan Ekuri, Jide Idris

**Affiliations:** 1 Department of Internal Medicine, College of Medical Sciences, University of Calabar, Calabar, Cross River State Nigeria; 2 National Aspergillosis Centre, University Hospital of South Manchester, Manchester, United Kingdom; 3 Department of Community Medicine, College of Medical Sciences , University of Calabar, Calabar, Cross River State Nigeria; 4 Department of Global Health and Population, Harvard T.H. Chan School of Public Health, Boston, Massachusetts USA; 5 Anadach Group, 2058 North Mills Ave, #447, Claremont, 91711 CA USA; 6 Anadach Consulting, 6B George Street, Lagos, Lagos State Nigeria; 7 Lagos State Ministry of Health, Lagos, Lagos State Nigeria

**Keywords:** Ebola virus disease, Nigeria, Lagos, Public health, Disease control

## Abstract

**Background:**

The 2014 Ebola virus disease (EVD) outbreak remains unprecedented both in the number of cases, deaths and geographic scope. The first case of EVD was confirmed in Lagos Nigeria on 23 July 2014 and spread to involve 19 laboratory-confirmed EVD cases. The EVD cases were not limited to Lagos State as Rivers State recorded 2 confirmed cases of EVD with 1 out of the 2 dying. Swift implementation of public health measures were sufficient to forestall a country -wide spread of this dreaded disease. This exploratory formative research describes the events of the Nigeria Ebola crisis in 2014.

**Methods:**

This research was implemented through key informant in-depth interviews involving 15 stakeholders in the EVD outbreak in Nigeria by a team of two or three interviewers. Most of the interviews were conducted face-to-face at the various offices of the respondents and others were via the telephone. The interviews which lasted an hour on average were conducted in English, digitally recorded and notes were also taken.

**Results:**

This study elucidated the public health response to the Ebola outbreak led by Lagos State Government in conjunction with the Federal Ministry of Health. The principal strategy was an incident management approach which saw them identify and successfully follow up 894 contacts. The infected EVD cases were quarantined and treated. The Nigerian private sector and international organizations made significant contributions to the control efforts. Public health enlightenment programmes using multimodal communication strategies were rapidly deployed. Water and sanitary facilities were provided in many public schools in Lagos.

**Conclusions:**

The 2014 Ebola outbreak in Nigeria was effectively controlled using the incident management approach with massive support provided by the private sector and international community. Eight of the confirmed cases of EVD in Nigeria eventually died (case fatality rate of 42.1%) and twelve were nursed back to good health. On October 20 2014 Nigeria was declared fee of EVD by the World Health Organization. The Nigerian EVD experience provides valuable insights to guide reforms of African health systems in preparation for future infectious diseases outbreaks.

## Background

The 2014 Ebola virus disease (EVD) outbreak remains unprecedented both in the number of cases, deaths and geographic scope [1]. The outbreak affected ten countries (Guinea, Liberia, Sierra Leone, Senegal, Nigeria, Mali, Spain, Italy, United Kingdom and the United States of America) in three continents (Africa, Europe & North America) killing over 11,300 people and infected over 28,000 [2]. The EVD was imported into Nigeria by a Liberian diplomat who arrived via Murtala Mohammed Airport Lagos on July 20, 2014. The diplomat had cared for a sibling with EVD in Liberia who eventually died from the disease on 8 July 2014 [3, 4]. He was identified as being unwell at the airport and admitted in a private hospital (hospital Z) in Lagos where the diagnosis of EVD was made on the third day. Millions of Nigerians were thrown into panic on July 23 2014 when the first case of EVD was confirmed in Lagos. On that same day, The Federal Ministry of Health in collaboration with the Nigeria Centre for Disease Control (NCDC), declared an Ebola emergency in Nigeria. With an estimated population of over 182 million people [5], Nigeria which is located in West Africa is the populous country in Africa characterized by a high degree of population movement across her borders. This high level of travel promotes the easy spread of infectious diseases [3]. Given that the EVD outbreak which began in December 2013 in Guinea had rapidly spread to Liberia and Sierra Leone, the risk of further spread to Nigeria was high. The unmitigated spread of EVD across West African countries was promoted by the fact that these countries had never experienced an EVD outbreak and were poorly prepared for this unfamiliar disease at every level [6].

The index EVD patient in Nigeria eventually died on July 25 2014 [7]. From this index case, the disease quickly spread to involve 19 laboratory-confirmed EVD cases and one probable case in two states. The World Health Organization (WHO) went on to declare EVD a Public Health Emergency of International Concern (PHEIC). This situation bears some similarity to the very recent Zika virus outbreak which has also been declared a PHEIC with the hope that this declaration will facilitate the mobilization of resources and promote a coordinated response to the growing threat [2]. Arguably, the lessons learnt from the EVD outbreak have accelerated the worldwide response to the Zika virus which is the first mosquito borne virus to be declared a PHEIC. Eight of the confirmed cases of EVD in Nigeria eventually died (case fatality rate of 42.1%) and twelve were nursed back to good health [8]. On October 20 2014 Nigeria was declared free of EVD by the WHO [9]. Despite the resounding success of the EVD control efforts in Nigeria, details of how this was achieved are yet to be fully chronicled.

## Methods

The aim of this study was to describe the events that occurred during the Nigeria Ebola crisis in 2014 with a view to identifying successes and gaps in the containment response.

### Setting of study

Lagos is the commercial nerve centre of West Africa with two domestic airports, an international airport and two seaports which have been adjudged to be the largest and busiest in the continent [10]. With a projected population of 22.58 million [11], Lagos has a total landmass of approximately 3345 km^2^ and a population density of about 20, 000 persons per square kilometre in the built-up areas [12]. Lagos was the first capital of Nigeria since its amalgamation in 1914 till the federal capital was moved to Abuja in 1991. Lagos Metropolitan Area consists of 16 Local Government Areas (LGAs). Lagos is a major economic hub in Nigeria and home to many financial institutions and major corporations. However, a significant proportion of Lagos residents live in slums without access to potable water and sanitation [13].

### Study design

This exploratory formative research was implemented through key informant in-depth interviews involving key stakeholders.

### Characteristics of study participants

Fifteen individuals who played major roles in the Nigeria EVD control efforts of 2014 were identified from analysis of existing reports on the EVD outbreak. These individuals were drawn from diverse backgrounds such as the government, non-governmental organizations (NGOs), hospitals and the private sector.

### Data collection and quality assurance

Fifteen in-depth interviews were held with the key informants by a team of two or three interviewers. Most of the interviews were conducted face-to-face at the various offices of the respondents and others were via the telephone. The interviews which lasted an hour on average were conducted in English, digitally recorded and notes were also taken. The digital recordings were transcribed verbatim by EA, SE and EO. The accuracy of the transcription was verified by repeatedly listening to the recorded interview while reviewing the transcripts.

### Analytical approach

The transcripts were thematically analysed by SA through reading and re-reading of the quotes. No qualitative software was used for the analysis. Using an inductive approach, SA assigned codes to emerging themes and developed a code book. These codes were verified by the co-authors through the reading and re-reading of the quotes. Differences in codes were discussed by co-authors until an agreement was reached. This process was used to verify the reliability of the data.

## Results

The key informants comprised of 10 males and 5 females with ages ranging from 35 to 65 years. Twelve of them were medical doctors. One of the doctors was an EVD survivor.

### Probable reasons for the importation of EVD into Nigeria

Key informants reported the lack of preparedness at the airports as a possible reason why the index case of EVD was imported into Nigeria.

“*Port health was not properly equipped. We were not fully aware of what to do at the time. Later, better knowledge of the disease brought about increased awareness. It was also as a result of shortage of personnel and diagnostic facilities*” [Health Commissioner]

### The index case (Mr. A)

A key informant reported that Mr. A, a Liberian government official, had arrived Lagos from Liberia. Mr. A was reportedly on his way to attend an ECOWAS (Economic Community of West Africa States) meeting in Calabar, Nigeria. He became unwell at the airport and was admitted in hospital Z [14]. At that time, there was a nation-wide strike by resident (postgraduate) doctors in Nigeria. Mr. A did not respond to treatment for malaria causing the lead physician to alert other colleagues. The diagnosis of EVD was made in three days and samples were also sent to the WHO reference laboratory in Dakar, Senegal, for confirmation [9].

*“On 20/7/2014, Mr. A (a delegate to an ECOWAS conference in Calabar) arrived at hospital Z vomiting. He tested positive for malaria and treatment was started on 21/7/2014. Later that day, he developed diarrhoea and started bleeding. By 22/7/14, Dr. G [lead physician] convened an incidence meeting having reached the conclusion that Mr A had an infectious haemorrhagic disease, most probably Ebola.”* [Dr H]

### Denial

Mr. A was reported to have repeatedly denied being in contact with an EVD patient while in Liberia where there was already an outbreak of EVD. He asserted that he had arrived Nigeria via Togo, a neighbouring country to Nigeria which had not yet recorded a case of EVD.

*“She [Dr. G] questioned him [index patient] and he denied contact with EVD patient(s). When she told him she suspected he had a haemorrhagic illness he asserted that he had come to Nigeria through Togo.”* [Dr I]

### Transfer of the index case to treatment centre stalled

Following the laboratory confirmation of EVD, Dr. G was reported to have had discussions with the Lagos State Ministry of Health about transferring the index case to the designated EVD treatment centre (hospital Y). However, this process was stalled because Dr. G was not impressed with the state of the treatment centre.

*“She (Dr G) said she had discussed with Dr. J about moving Mr A to hospital Y. She [Dr. G] was not impressed with the state of hospital Y after inspecting it and refused to move Mr. A stating: 'You cannot keep an animal in there.'”* [Dr I]

### Pressure to release the index case resisted

A key informant reported there was immense pressure from some Liberian government officials for Dr. G to release Mr. A from hospital Z. Dr. G responded by arranging for a barrier to be put on Mr. A’s door to refrain him from leaving.

*“On 22/7/2014, a Liberian government official wanted Mr. A released to attend a meeting in Calabar. Dr. G was told that Mr. A’s human rights were being violated. Dr. G insisted that Mr. A be kept in for the greater public good, and asked for a barrier to be put on his door.”* [Dr I].

The position taken by Dr. G to quarantine Mr. A was supported by the Medical Director of hospital Z despite threats of legal action against the hospital by the Liberian Embassy [3]. The Lagos State Government reportedly responded to these pressures by not only refusing to release Mr. A, but cremating his remains on the day he died. The Lagos State Government had previously enacted a cremation law in April after which a crematorium was established in hospital Y [7].

*“The Lagos State Government was firm in its decision to refuse the request to return the body of Mr. A (index case) to Liberian authorities as this was perceived to be a public health hazard. The body was subsequently cremated”* [Dr F]

### Political will and commitment

The Lagos State Government, led by the Governor and Health Commissioner instituted control measures. Huge sums of money were said to have been made available to manage the EVD outbreak. Monetary incentives were provided by the Lagos State Government for medical volunteers in hospital Y. Comprehensive life insurance policies were quickly drawn up for all medical volunteers [7].

*“The Governor of Lagos State co-ordinated the outbreak control activities. The Health Commissioner as well was always on the field”* [Prof Q]

*“An estimated 1 billion naira was spent by the Lagos State Government while the Federal Government contributed 200 million”* [Medical Director].

*“In the first 10 days, only one WHO staff was admitting patients in the treatment centre as Nigerian health workers were very reluctant to volunteer. Finally, the Lagos State Government was able to get volunteers using a reward system.”* [Medical Director].

### Response to the EVD outbreak

The Emergency Operations Centre (EOC) was set up by the Lagos State Government. It consisted of six different units. In this article, the response to the EVD outbreak has been categorized into primary, secondary and tertiary measures. Hospital Y was designated as the EVD treatment centre.

*“The Lagos State Government set up a rapid response team which later became the EOC which comprised 6 teams…. The EOC was the key in the intervention process which guaranteed control and planning”* [Dr L].

#### Primary prevention

The Lagos State Government had embarked on extensive community enlightenment prior to the outbreak. This helped to douse the tensions that built up during the EVD outbreak.

*“Four months before the outbreak, Lagos State had already sensitized the public through print and electronic media about the spread of Ebola. In depth interviews had been done on television and radio stations. The importance of hand hygiene and environmental hygiene was highlighted.”*[Dr. M]

Multimodal communication strategies were deployed to reach the general population. A special unit had to be created to manage information on EVD in Lagos State.

*“Tools used included social media, bulk Short Messaging Service (SMS), Ebola Alert; mainstream media. They all helped debunk misconceptions.”* [Dr. L]

*“An information unit was created and dedicated to the purpose of massive media publicity including online (social media) - Ebola alert. An Ebola helpline was also created.”* [Health Commissioner].

At the time the index case was reported, primary and secondary schools in Lagos State were on holiday. In order to circumvent the spread of EVD, the State Government was said to have delayed resumption of these schools. Potable water and toilet facilities were also provided in public schools across Lagos [7].

*“School resumption was delayed while the state government supplied the schools with hand hygiene kits in preparation for re-opening.”* [Medical Director]

Following laboratory confirmation of the index case, a list of contacts of the index case was drawn up.

*“In accordance with Center for Disease Control and Prevention protocol, Dr. G (the attending physician of the index patient) started drawing up contacts of Mr. A.”* [Dr H]

These contacts who did not manifest symptoms of EVD were followed up in their homes as they were deemed non contagious.

*“For contacts of confirmed cases, the approach was to follow them up at home. It was safe to leave them at home as long as they were not manifesting symptoms. Disease notification officers in each Local Government Area collaborated with the EOC in contact tracing. Approximately 95% of contacts were traced.”* [Medical Director]

Wide spread publicity, public awareness and fear of the contagion among the general populace contributed to the prevention of further spread of the disease.

*“The fear of the contagion and the fact that Lagos State people are a listening people also helped a great deal”* [Prof. M]

There were concerns by people living within the vicinity of hospital Y about potential contamination arising from cremation of the bodies of the EVD victims. Community enlightenment activities were deployed to dispel these fears.

*“The community living around hospital Y was worried about getting infected by Ebola particles (from the crematorium).”*[Dr N].

### Secondary prevention

The patients who were diagnosed with EVD were transferred to hospital Y. This facility was only hurriedly converted to an EVD treatment centre.

*“Confirmed cases were immediately confined to the treatment centre”* [Medical Director]

### Tertiary prevention (rehabilitation)

Although hospital Z was shut down during the EVD outbreak to prevent the spread of infection, the staff were still being paid salaries from funds provided to the hospital by the Lagos State Government. The children of some patients who died from EVD were reportedly ejected from their homes due to stigma. Such children were provided new homes.

*“Hospital Z was shut down for two months… Salaries of staff were still being paid during those two months. The children of late X [who died from EVD] were driven out of their home as a result of stigma. The children now have a new home, and they are back in school.”* [Dr. H]

Survivors of the EVD were said to have been offered monetary incentives by the Lagos State government to compensate for job losses due to stigma. The survivors and their families were also reported to have been offered counselling and psychosocial support.

*“Monetary incentives to survivors and families of the dead were provided. There was also employment for survivors as well as psychosocial and counselling support.”*[Health Commissioner].

### Partnerships

#### International community participation

Nigeria benefitted immensely from support from the international community and the EOC coordinated this.

*“The EOC consisted of six teams:* *A) Epidemiology and Surveillance: supported by the WHO.* *B) Case Management and Infection control: supported by Medecins Sans Frontieres (MSF), the WHO and Lagos State Ministry of Health (LSMOH)* *C) Social Mobilization: supported by United Nations Children’s Fund (UNICEF).* *D) Laboratory Services: supported by CDC and WHO.* *E) Point of Entry: supported by WHO, CDC, and UNICEF.* *F) Management and Coordination: supported by UNICEF and Center for Disease Control and Prevention (CDC).*”[UNICEF representative]

ᅟManagement of data was handled professionally by E-health, an international NGO working on eradicating polio in Nigeria.

*“E-health was responsible for data management and provided data enabled phones, Global Positioning System (GPS) and household survey materials. They were also in charge of the server used by the response team.”*

[UNICEF representative]

#### Public-private partnerships

The Lagos State Government initiated efforts to partner with the private healthcare practitioners.

*“… AGPMPN (Association of General and Private Medical Practitioners of Nigeria) always has regular meetings with HEFAMAA [Health Facility Monitoring and Accreditation Agency]. So it was easy to organise the private health sector during the outbreak through this group.”* [Mrs. T].

Private business concerns also made substantial contributions to the EVD control efforts.

*“The Dangote foundation provided NGN 152,956,250.00 for setting up the EOC, as well as the salaries of staff and volunteers for 6 months. The foundation also provided 12 units of thermal scanning systems for the 4 international airports in Nigeria. In addition, the foundation sponsored the training of 160 staff of Federal Ministry of Health and Port Health Services at a total cost of NGN 66,657,270.00. The foundation also donated 3,800 sets of personal protective equipment costing NGN 25,938,753.63 to the Nigerian Centre for Disease Control and covered the costs (NGN 60,000,000.000) of quarantining the returnee Nigerian volunteer delegation.”* [Mr. Z]

*“Total and Oando (private companies) set up an education trust fund for children of the deceased” [Dr. M]*

*“Chevron and Mobil gave 3 ambulances, Total donated 5 Hilux vehicles…” [Dr. N]*

### Faith-based organizations

The Lagos State Government reportedly tried to dissuade two churches with large membership from hosting international conferences during the EVD outbreak.

*“There were rumours at the time of the outbreak that two churches were convening international conferences. Letters were written to warn them to suspend these. However, one of them, Church A had already begun. They assured the authorities that infection control measures had been put in place in the church. Church B was contacted and visited by the Commissioner and his team”* [Health Commissioner]

### The media

The media was reported to have played a very critical role.

*“A lot of information went out. It involved all strategies to communicate with the people. Virtually all media houses came forward to get accurate information on Ebola from the Lagos State Ministry of Health .Information on Ebola was rife.”*[Mrs T]

### The spread to Rivers State

A contact of the index case was isolated at home after blood samples were taken for EVD testing. Unfortunately, the contact absconded to Rivers State and placed himself under the care of a doctor. This doctor decided to treat the contact in his hotel room and died in the process. Blood samples from the both the contact and the doctor finally tested positive for EVD. The doctor was in turn linked to a total of 526 contacts in Port Harcourt [3]. Rivers State eventually managed 8 suspected EVD cases and 2 confirmed cases with 1 out of the 2 dying.

*“One of the contacts went off to Port Harcourt (Rivers State) and another to Enugu.”*[Medical Director]

### Challenges

Several difficulties were experienced during the EVD outbreak. The first few days proved to be very chaotic.

*“There was panic in all quarters; there was confusion even at the Federal Government level.”* [Dr. L].

A lack of infrastructure was reported to be one of the greatest challenges faced. The decision to convert hospital Y to an EVD treatment centre was made only after the index case was diagnosed. There were no quarantine centres; contacts were traced and monitored in their homes.

*“A big challenge was lack of a standard quarantine station. The decision to use hospital Y as a treatment centre was a late one ”* [Medical Director]

Another challenge experienced was the on-going medical doctors’ strike in public health facilities at the onset of the outbreak

*“The doctors’ strike was a major problem during the outbreak because it made it difficult* *to convince the international community we needed help when our doctors were on strike” [Dr. H]*

The industrial action by the doctors was eventually resolved following the intervention of Nigerian doctors in diaspora.

“The Association of Nigerian Physicians in America (ANPA) in the United States and other notable officers banded together to put pressure on NMA (Nigerian Medical Association) to call off the strike.” [Dr. H]

The EVD outbreak is reported to have impacted negatively on the commercial activities of Lagos State [15]. The Lagos State hospitality industry reportedly lost over 8 billion Nigerian Naira between July and October 2014 due to plummeting hotel occupancy rates [3].

### Implications and lessons learned

#### Health system’s emphasis on curative medicine

Key informants reported that the EVD outbreak revealed how unprepared and vulnerable the health system was. It was also reported that the health system was suited for curative rather than preventive medicine.

*“The outbreak has revealed how vulnerable and weak our public health infrastructure is.”* [Health Commissioner]

*“The outbreak highlights the need for greater emphasis to be placed on prevention rather than treatment in our health care system.”* [Dr. F]

### Exporting human resources to EVD-affected West African sub-regions

About 250 health personnel who had been trained in Nigeria received further training and travelled under the aegis of African Union – African Support to Ebola Outbreak in West Africa (AU-ASEOWA) to render services to other EVD-affected West Africa sub-regions.

*“I was one of the facilitators of the training of 250 personnel who went on to combat EVD in some neighbouring countries under the AU-ASEOWA initiative”* [Dr. F]

### Multi-sectorial approach to crisis management

The need for an emergency response framework was very evident during the EVD outbreak in Nigeria.

“The response team comprised of several groups but there was no formal working agreement which brought about problems of ownership, accountability and working limits.”

[UNICEF representative]

*“LASEMA (Lagos State Emergency Management Authority) the ambulance and fire services were also instrumental. LAWMA (Lagos State Waste Management Agency) helped with disposal of hospital waste.”* [Health Commissioner]

The uncertainty of stakeholders with respect to their specific roles was eventually overcome. A solid structure is now in place in Lagos State to facilitate her response to future emergencies.

*“The EOC is still functional and meets once a week.”*

[UNICEF representative]

*“Infection prevention and control activities are still ongoing as well as preparedness training in all the states in Nigeria.”*[Mrs T]

There are also plans to upgrade facilities in hospital Y to position it to respond efficiently to future infectious diseases outbreaks.

*“All hospitals are to embark on training and re-training programmes. There is a plan underway to build up hospital Y to be able to quarantine and treat infectious disease cases*.” [Prof M]

## Discussion

The EVD outbreak in West Africa has come and gone leaving in its wake devastated families and communities. The deaths recorded in this particular outbreak are said to surpass those recorded during the previous 25 Ebola epidemics combined [16]. The lack of infectious disease surveillance capacity in West Africa was identified as a major factor that contributed to the spread of EVD in this region which had never experienced an EVD outbreak [17]. The concept of “an old disease in a new context” favoured the rapid and almost invisible spread of EVD [18]. In Nigeria, the rapid control of the EVD was facilitated by the rapid detection of the index case, the comprehensive contact tracing measures and the isolation and treatment of the secondary cases [3, 19]. In Guinea, Liberia and Sierra Leone, EVD diagnosis of EVD was only made after several weeks thereby allowing for spread of the disease among the population.

Amidst all the uncertainties that characterized the first few days of the EVD response in Nigeria, the Lagos State Government provided the vital leadership in conjunction with the Federal Ministry of Health. Using the incident management approach which was coordinated by the EOC, the massive support provided by the private sector and international community was effectively harnessed. With respect to contact tracing, a total of 894 contacts were identified and approximately 18,500 face-to-face visits were conducted to assess contacts for symptoms of EVD. Flight manifests and phone records were reportedly used in the contact tracing exercise [20].

The isolation and treatment of secondary cases was initially challenging as there were no isolation facilities. Considering the relatively common occurrence of infectious disease outbreaks in African settings, the provision of such isolation facilities and training of healthcare personnel needs to be given priority. It is reported that even before the Nigerian EVD outbreak, specialists from the Centers for Disease Control and Prevention (CDC) (GA, USA) had already begun training one hundred physicians on epidemiology [21]. This training might have positioned the country to provide a robust response the EVD outbreak [22].

Social mobilization using multimodal strategies was very evident in Nigeria’s response to the EVD. The effectiveness of disseminating educational materials via electronic channels such as Twitter and Facebook has been reported severally [23–26] and the EVD outbreak in Nigeria mirrors this. Given that the use of electronic communication devices has become commonplace even in resource poor settings, disease control strategies need to make full use of this resource to drive behaviour change during outbreaks. This was vital in the Nigerian outbreak as rumours were rife that *Garcinia kola* (bitter kola) and salt could cure EVD [27, 28].

The EVD outbreak in Nigeria recorded significant gains from a public heath perspective as water and sanitary facilities were provided in many public schools in Lagos and other parts of the country. It is important that desirable habits such as hand washing should continue as this singular action can reduce the incidence of other infectious diseases such as diarrhoea.

## Conclusion

The 2014 Ebola outbreak in Nigeria was effectively controlled using the incident management approach with massive support provided by the private sector and international community. As the African subcontinent recovers from the onslaught of EVD, it is time to reposition her health system to be able to effectively contain future disease outbreaks. The Nigeria EVD experience provides valuable insights to guide these vital reforms.

## Abbreviations

AGPMPN: Association of General and Private Medical Practitioners of Nigeria
ANPA: Association of Nigerian Physicians in America
AU-ASEOWA: African Union – African Support to Ebola Outbreak in West Africa
CDC: Center for disease control and prevention
Dr. F: Physician in a state tertiary health facility and member of the EOC response team
Dr. G: Lead physician in the private specialist facility
Dr. H: Physician in the private specialist facility
Dr. I: A relative of Dr. G
Dr. J: Lagos State Ministry of Health Official and key player in the EVD response
Dr. K: NCDC representative and member of EOC response team
Dr. L: Physician and member of EOC response team
Dr. M: Lagos State Ministry of Health Official and EOC team lead
Dr. N: NFELTP representative and member of EOC response team
ECOWAS: Economic Community of West African States
EOC: Emergency operations centre
EVD: Ebola virus disease
GPS: Global positioning system
HEFAMAA: Health facility monitoring and accreditation agency
Hospital Y: the EVD treatment centre
Hospital Z: Private specialist health facility
LASEMA: Lagos State emergency management authority
LAWMA: Lagos State waste management agency
LSMOH: Lagos State Ministry of Health
Mr. A: the index case
Mr. Z: Private sector representative
Mrs. T: Lagos state ministry of health representative
NCDC: Nigeria centre for disease control
NGO: Non-governmental organization
NMA: Nigerian medical association
PHEIC: Public health emergency of international concern
Prof. M: Consultant physician in a state tertiary health facility
Prof. Q: Infectious disease specialist in a federal tertiary health facility and EOC team lead
SMS: Short messaging service
UNICEF: United Nations Children’s Fund
WHO: World Health Organization
X: an EVD case who died
